# Supplementary material for: Complexes of Cu–Polysaccharide of a Marine Red Microalga Produce Spikes with Antimicrobial Activity
Source: Mar Drugs. 2022 Dec 19;20(12):787. doi: 10.3390/md20120787 (PMC9783634; doi:10.3390/md20120787)
Supplement: Supplementary file 1 [file marinedrugs-20-00787-s001.zip › marinedrugs-2032233-supplementary.pdf]

Supporting Information

Complexes of Cu-Polysaccharide of a Marine Red Microalga Produce Spikes with Antimicrobial Activity

Nofar Yehuda, Levi A. Gheber, Ariel Kushmaro and Shoshana (Mails) Arad*

*Corresponding author: arad@post.bgu.ac.il
Department of Biotechnology Engineering
Room 126 Bldg. 42, P.O.B 653, Beer-Sheva 8410501, Israel

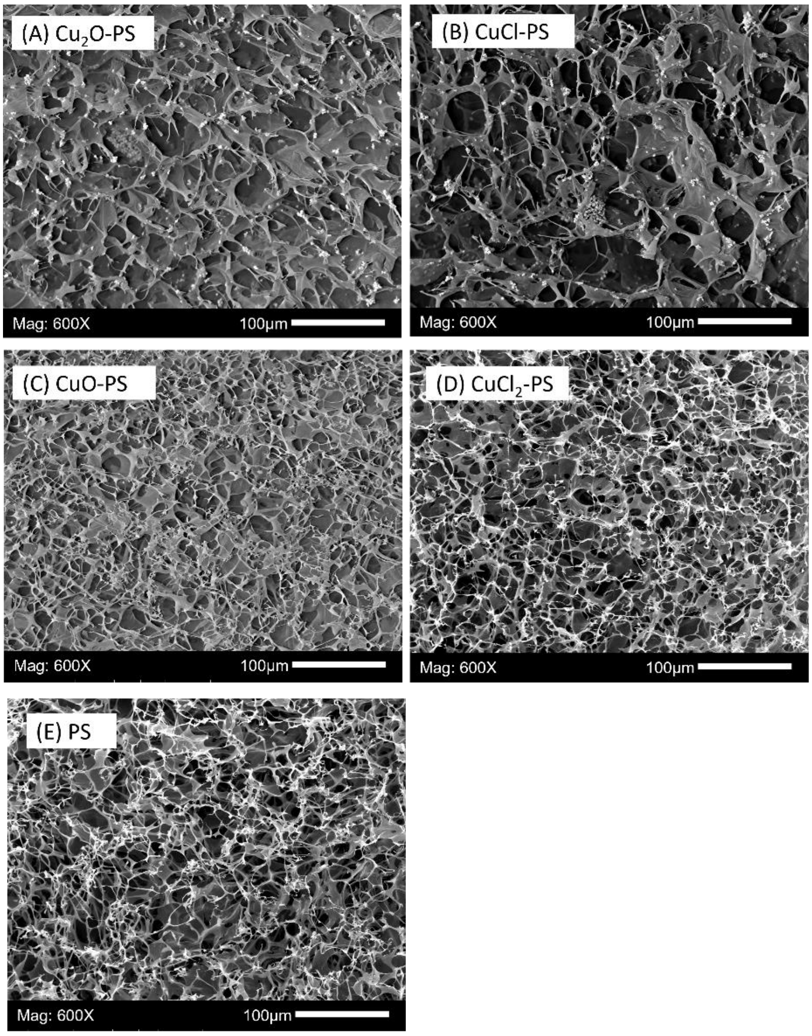
(A) Cu_2O-PS
Mag: 600X
100µm
(B) CuCl-PS
Mag: 600X
100µm
(C) CuO-PS
Mag: 600X
100µm
(D) $CuCl_2$-PS
Mag: 600X
100µm
(E) PS
Mag: 600X
100µm

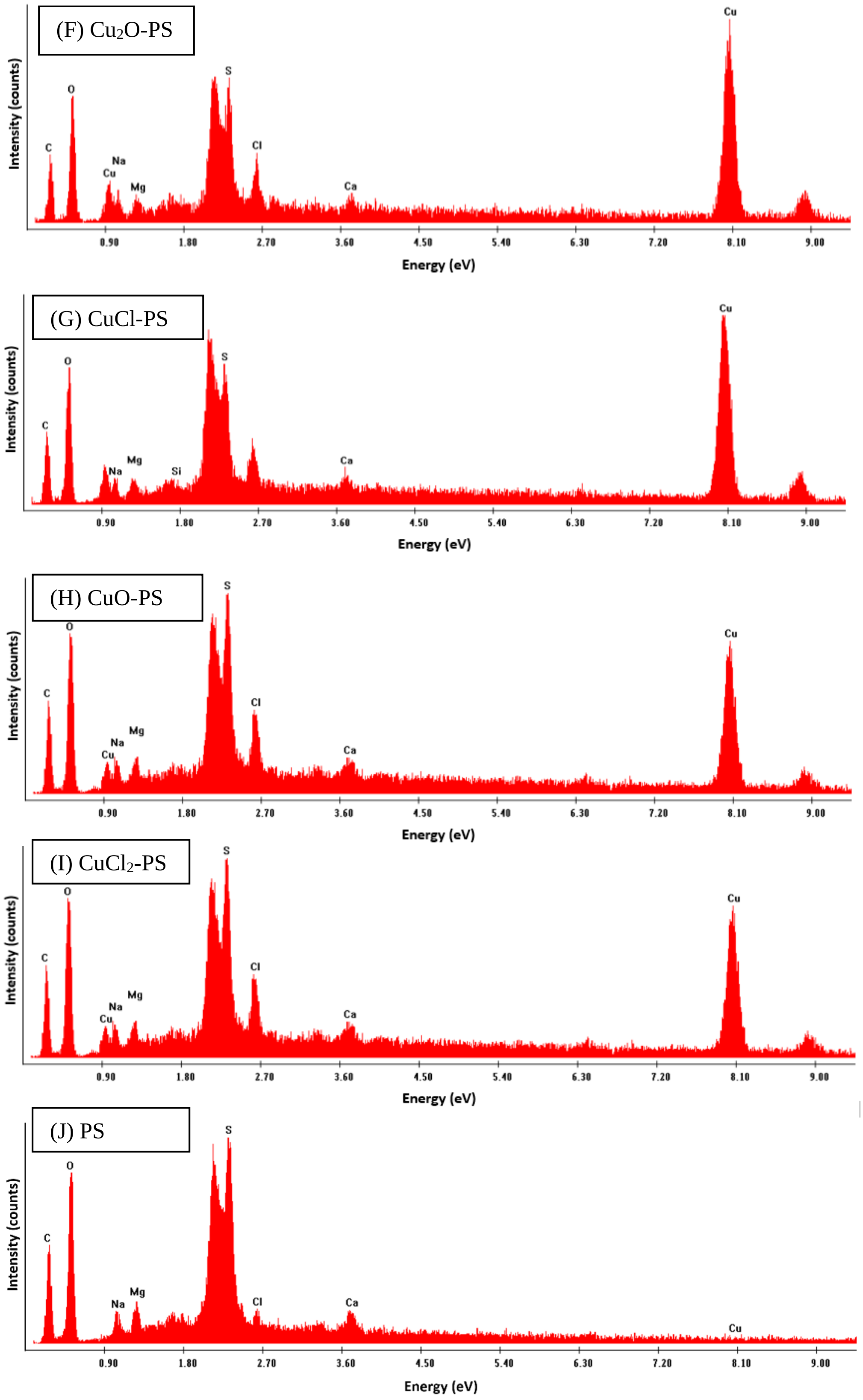

Figure S1. SEM micrographs (A-E) and EDS spectra of the Cu-PS complexes and PS (F-J). The samples were freeze-dried and coated with gold. ×600. All the Cu−PS complexes contained 0.7% (w/v) polysaccharide and 500 ppm copper.

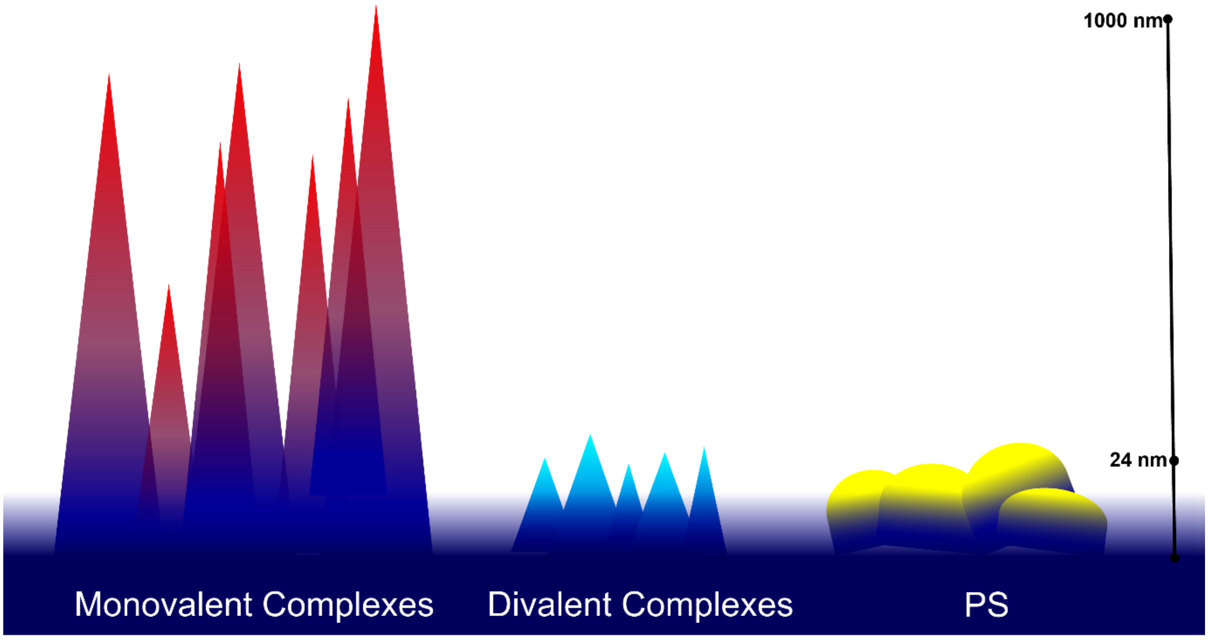

Figure S2. Schematic illustration of spikes from monovalent (red) and divalent (blue) Cu-PS complexes and PS (yellow) - transect.

		C. albicans	*A. baumannii*	*P. aeruginosa*	*E. coli*	*S. aureus*	*B. subtilis*
		a) Growth Inhibition [%]					
Monovalent	Cu_2O-PS	92.8±0.1	78.5±9.8	78.1±5.1	75.0±1.5	72.9±7.1	79.3±2.5
	CuCl-PS	88.9±2.3	69.4±2.7	66.7±2.3	75.8±2.9	75.9±3.2	83.4±5.9
Divalent	CuO-PS	21.2±5.7	48.4±5.9	40.2±6.0	59.1±5.3	38.9.±32	35.7±4.2
	$CuCl_2$-PS	48.7±0.1	45.9±4.1	44.8±3.5	66.4±1.2	34.3±8.9	35.3±2.0
Controls	PS	12.2 ± 1.7	32.4 ± 3. 6	38.6 ± 8.3	49.2 ± 4.1	30.8 ± 5.6	29.6 ± 2.9
	Cu_2O	4.7 ± 0.1	22.4 ± 4.9	21.5 ± 9.4	15.2 ± 2.0	20.9 ± 8.9	10.6 ± 5.8
	CuCl	2.3 ± 1.6	5.6 ± 3.5	4.1 ± 4.2	7.8 ± 2.9	6.5 ± 3.4	10.5 ± 4.5
	CuO	2.5 ± 0.3	4.5 ± 5.6	9.7 ± 2.8	1.6 ± 0.9	9.8 ± 4.8	8.5 ± 2.5
	$CuCl_2$	1.8 ± 0.7	7.8 ± 6.5	7.8 ± 4.1	4.3 ± 2.0	8.9 ± 5.2	9.6 ± 5.6
		b) Cell Viability [%]					
Monovalent	Cu_2O-PS	5.6 ± 0.5	4.6 ± 0.8	3.6 ± 1.3	3.8 ± 2.3	2.0 ± 0.3	1.0 ± 0.9
	CuCl-PS	11.7 ± 0.6	6.7 ± 0.4	5.4 ± 2.9	7.9 ± 4.5	8.7 ± 2.5	4.7 ± 2.5
Divalent	CuO-PS	36.0 ± 5.3	22.3 ± 4.5	25.6 ± 2.3	27.7 ± 5.4	31.7 ± 4.9	21.7 ± 2.9
	$CuCl_2$-PS	31.7 ± 1.5	28.4 ± 0.8	30.7 ± 4.1	37.4 ± 4.5	31.2 ± 4.1	42.3± 3.1
Controls	PS	55.0 ± 8.7	35.4 ± 2.3	36.8 ± 0.8	38.7 ± 0.7	32.4 ± 3.7	35.6 ± 3.6
	Cu_2O	61.0 ± 1.7	32.4 ± 4.5	35.4 ± 2.6	37.2 ± 5.4	41.6 ± 6.5	34.7 ± 7.8
	CuCl	90.3 ± 0.6	37.4 ± 3.5	38.9 ± 4.5	40.9 ± 8.4	42.4 ± 2.3	52.6 ± 5.4
	CuO	84.7 ± 4.0	87.1 ± 2.3	79.4 ± 8.9	78.1 ± 6.5	71.2 ± 8.9	72.7 ± 4.0
	$CuCl_2$	71.0 ± 1.0	81.5 ± 7.4	82.6 ± 6.8	81.3 ± 5.6	81.6 ± 4.5	82.0 ± 1.0

Table S1. Effect of the mono- and divalent Cu-PS complexes on the (a) inhibition of growth and (b) cell viability of fungi and Gram-negative and Gram-positive bacteria. All the Cu-PS complexes and controls contained 0.07% (w/v) polysaccharide and 30 ppm copper. For the growth inhibition experiment, the control was the absorbance of only LB or TSB plus bacteria or PDB plus fungus. The microbial cultures were incubated with shaking in 96-well plates at 37 °C for 14 h for *A. baumannii*, *P. aeruginosa* PA14, *E. coli*, *S. aureus* and *B. subtilis* or 48 h for *C. albicans*. Each sample was plated on an LB, TSB or PDB agar plate, as relevant, and after serial dilution incubated overnight at 37 °C. On the following morning, CFUs were counted. CFU values were determined in comparison with the control (untreated cells), and cell viability was calculated. Values are means ± SD of three independent experiments performed in triplicate.

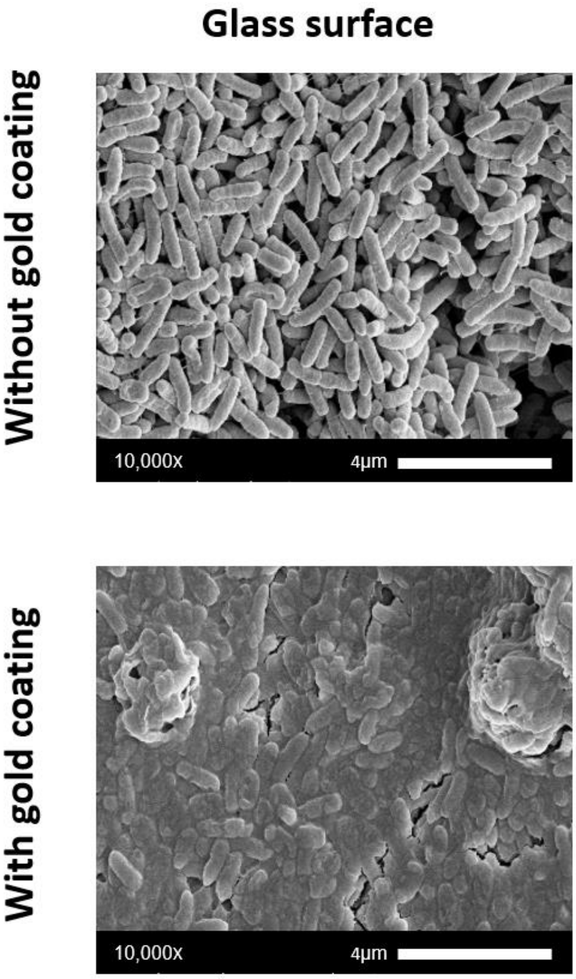

Figure S3. *P. aeruginosa* PA14 biofilm formation on glass surfaces with (lower image) and without (upper image) gold coating. ×10,000, scale bar=4μm.

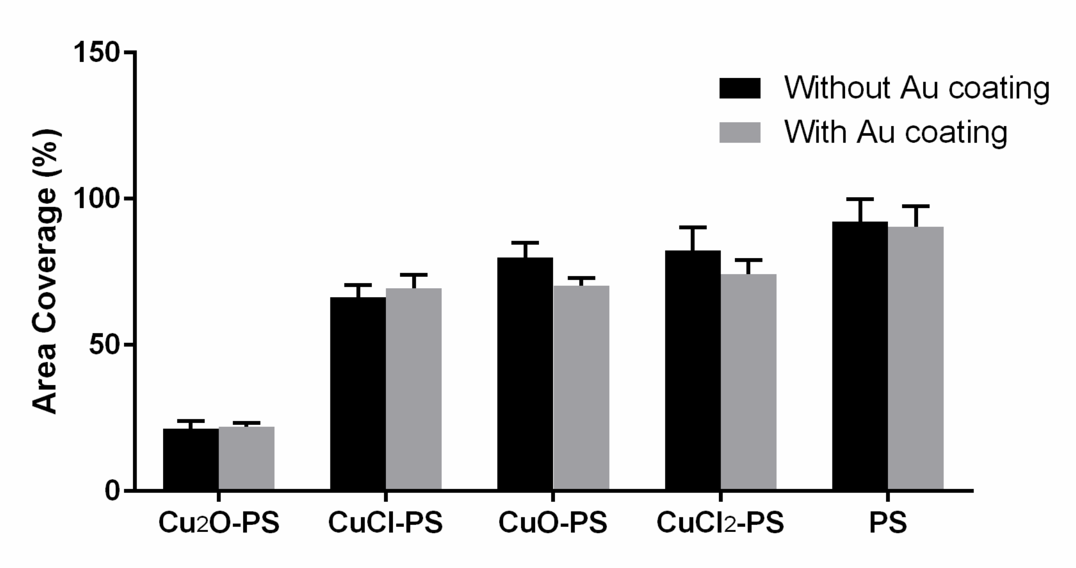

Figure S4. Effect of monovalent and divalent Cu-PS complexes that were surface coated with gold (black columns) or non-coated (gray columns) on *P. aeruginosa* PA14 biofilm formation. Bacterial quantification of the SEM images from Fig. 7. All the Cu-PS complexes contained 0.7% polysaccharide (w/v) and 500 ppm copper.

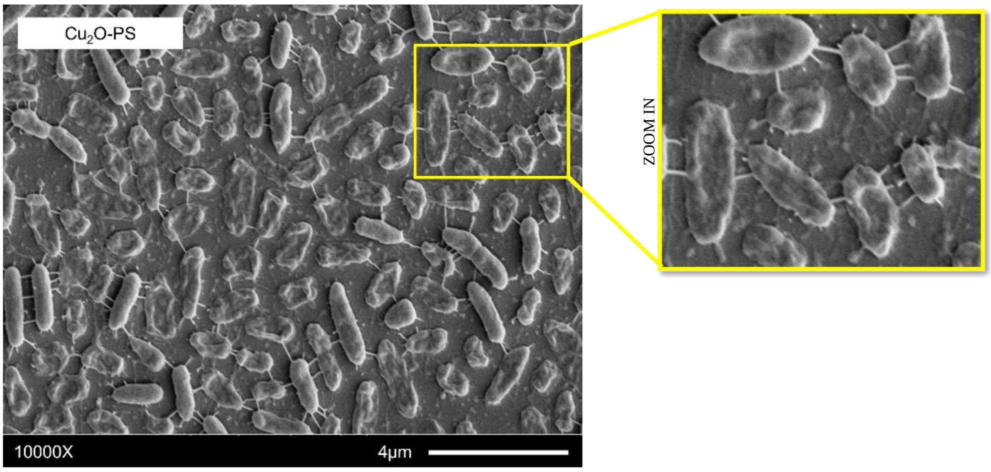

Figure S5. SEM micrographs of *E. coli* TV1061 treated with the Cu_2O-PS complex. ×10,000, scale bar=4µm.

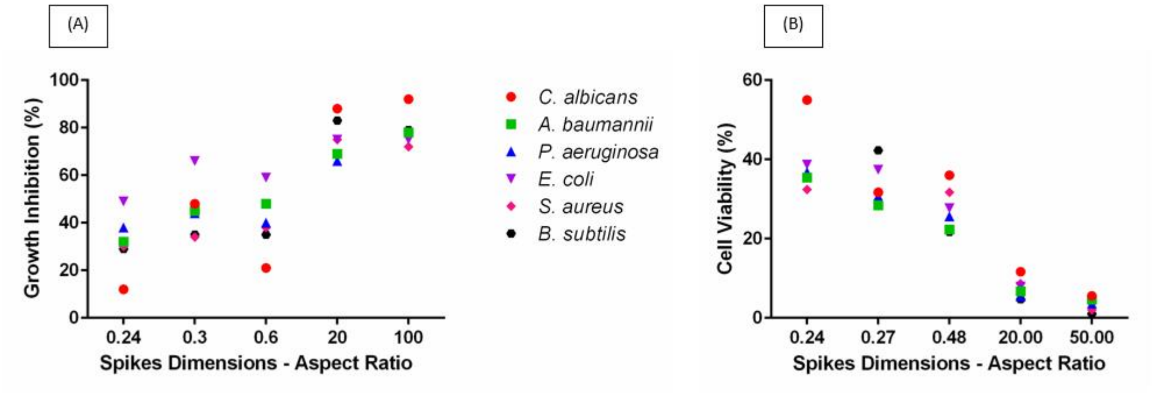

Figure S6. Microbial inhibition (A) and cell viability as a function of spike dimensions (aspect ratio) for the following microorganisms: *Candida albicans* (red), *Acinetobacter baumannii* (green), *Pseudomonas aeruginosa* (blue), *Escherichia coli* (purple), *Staphylococcus aureus* (pink) and *Bacillus subtilis* (black). All the Cu−PS complexes contained 0.07% (w/v) polysaccharide and 30 ppm copper. The results presented are calculated from Fig. 3 and Fig. 6.
